# Detection of Few Hydrogen Peroxide Molecules Using Self-Reporting Fluorescent Nanodiamond Quantum Sensors

**DOI:** 10.1021/jacs.2c01065

**Published:** 2022-06-23

**Authors:** Yingke Wu, Priyadharshini Balasubramanian, Zhenyu Wang, Jaime A. S. Coelho, Mateja Prslja, Reiner Siebert, Martin B. Plenio, Fedor Jelezko, Tanja Weil

**Affiliations:** † Max Planck Institute for Polymer Research, Ackermannweg 10, 55128 Mainz, Germany; ‡ Institute for Quantum Optics and IQST, Ulm University, Albert-Einstein-Allee 11, Ulm 89081, Germany; § Institute of Human Genetics, Ulm University and Ulm University Medical Center, Ulm 89081, Germany; ∥ Institut für Theoretische Physik und IQST, Universität Ulm, Albert-Einstein-Allee 11, Ulm 89081, Germany; ⊥ Guangdong Provincial Key Laboratory of Quantum Engineering and Quantum Materials, School of Physics and Telecommunication Engineering, South China Normal University, Guangzhou 510006, China; # Guangdong-Hong Kong Joint Laboratory of Quantum Matter, Frontier Research Institute for Physics, South China Normal University, Guangzhou 510006, China; ¶ Centro de Química Estrutural, Institute of Molecular Sciences, Faculty of Sciences, University of Lisbon, Campo Grande, Lisbon 1749-016, Portugal; ∇ Institute of Inorganic Chemistry I, Ulm University, Albert-Einstein-Allee 11, Ulm 89081, Germany

## Abstract

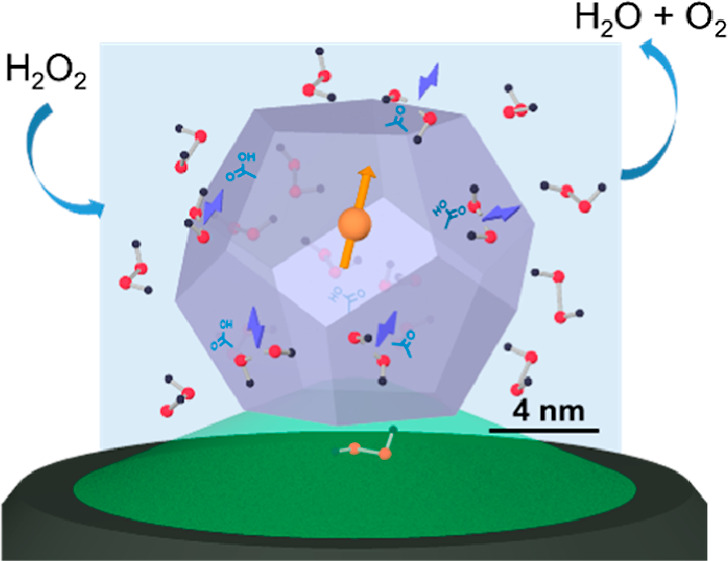

Hydrogen peroxide (H_2_O_2_) plays an important role in various signal transduction pathways and regulates important cellular processes. However, monitoring and quantitatively assessing the distribution of H_2_O_2_ molecules inside living cells requires a nanoscale sensor with molecular-level sensitivity. Herein, we show the first demonstration of sub-10 nm-sized fluorescent nanodiamonds (NDs) as catalysts for the decomposition of H_2_O_2_ and the production of radical intermediates at the nanoscale. Furthermore, the nitrogen-vacancy quantum sensors inside the NDs are employed to quantify the aforementioned radicals. We believe that our method of combining the peroxidase-mimicking activities of the NDs with their intrinsic quantum sensor showcases their application as self-reporting H_2_O_2_ sensors with molecular-level sensitivity and nanoscale spatial resolution. Given the robustness and the specificity of the sensor, our results promise a new platform for elucidating the role of H_2_O_2_ at the cellular level.

## Introduction

Reactive oxygen species (ROS) are highly reactive molecules such as free radicals formed from molecular oxygen. One of the key ROS is hydrogen peroxide (H_2_O_2_), which is produced in cells during oxygen metabolism. Compared to the highly reactive hydroxyl radical, whose reported half-life within cells is about 1 ns,^1^ the less reactive H_2_O_2_ is involved in various physiological processes such as hypoxic signal transduction, cell differentiation, proliferation, migration, and apoptosis.^2^ The influence of H_2_O_2_ is particularly dependent on its location and concentration.^3^ For example, H_2_O_2_ exhibits either pro- or anti-apoptotic functions depending on its localization and intracellular concentration.^2^ Moreover, H_2_O_2_ also acts as a biomarker in various human diseases,^4^ such as Alzheimer’s disease,^5^ cardiovascular diseases,^6^ and cancer.^7^ Cancer cells can maintain a higher H_2_O_2_ and an impaired redox balance, thereby affecting the tumor microenvironment and the antitumor immune response.^2^ Elucidating the role of H_2_O_2_ in biological systems is still limited by low analyte concentrations and the short lifetime within cells with a reported half-life of about 1 ms.^1,8^ Over the past few years, various H_2_O_2_ selective probes have been developed, including fluorescence-based small molecules/polymers,^9,10^ electrochemiluminescence approaches,^11,12^ optical sensors,^13^ and positron emission tomography.^14^ However, detecting a few H_2_O_2_ molecules with high sensitivity and spatial resolution at the nanoscale remains a challenge.

Nanodiamonds (NDs) with negatively charged nitrogen-vacancy (NV^–^) centers have received much attention as promising emitters and sensors for biological applications.^15^ Recently, fluorescent NDs have extensively been used as so-called quantum sensors for detecting various physical parameters such as magnetic field,^16^ temperature,^17,18^ and pH.^19^ The exceptional photostability of fluorescent NDs combined with the opportunity to attach various surface groups and the biocompatibility of the material^20^ makes them well suited for biological applications^21^ such as single particle tracking,^22^ nanothermometry,^23,24^ and magnetic imaging.^25,26^ These nanosensors can be used to detect a few external paramagnetic spins by measuring the effects of the magnetic noise produced by the electron spins on the *T*_1_ relaxation time of the NV centers. So far, *T*_1_ relaxometry has been used for the detection of a range of paramagnetic spins such as gadolinium,^27^ ferritin,^28^ and most recently free radicals.^29,30^ However, *T*_1_ relaxometry is insensitive to non-paramagnetic species such as H_2_O_2_.

**Scheme 1:**
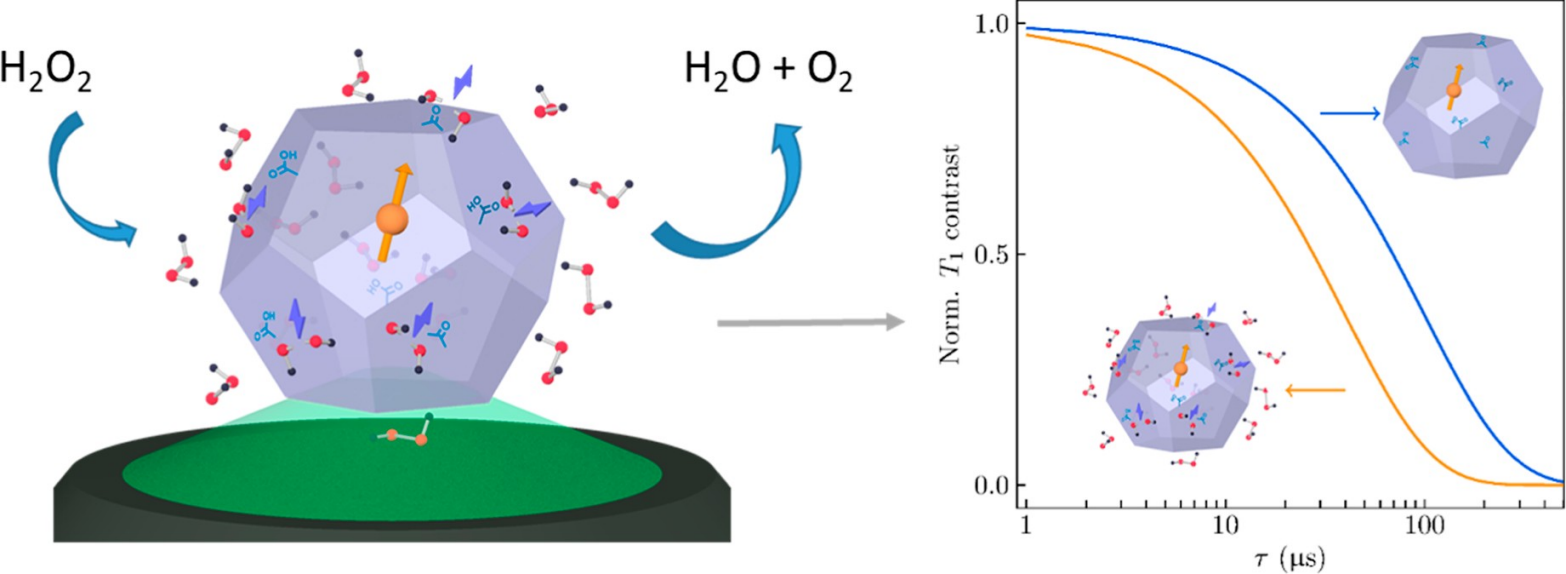
Structure of a Self-Reporting Peroxidase-like ND Sensor for H_2_O_2_ Detection

Recent studies have shown that oxygenated detonation NDs exhibit peroxidase-mimicking functionalities, forming radicals as intermediates due to their ultra-small size (less than 5 nm) and the distorted oxygen-containing groups on the surfaces.^31,32^ In this work, we present for the first time the ultrasensitive self-reporting H_2_O_2_-sensing properties of oxygenated fluorescent NDs produced by the high-pressure high-temperature method due to their peroxidase-mimicking activities and quantum property (Scheme 1). This enables us to reveal the spatiotemporal distribution of H_2_O_2_ local concentrations and their constant changes determined by numerous local processes of peroxide formation and elimination in living cells. In contrast, current methods only allow a rough assessment of the average basal H_2_O_2_ level and its fluctuations in living cells.^33^ First, we prove the peroxidase-mimicking activities of 10 nm oxygenated florescent NDs using 3,3′,5,5′-tetramethylbenzidine (TMB) as a colorimetric indicator. Furthermore, we use density functional theory (DFT) calculations to mechanistically elucidate the role of the diamond surface groups in the decomposition of H_2_O_2_ molecules. We also showcase NV centers as nanoscale sensors for detecting intermediate radicals in the catalytic decomposition of H_2_O_2_ by measuring the effects of the magnetic noise produced by the radicals on the *T*_1_ time of the NV centers. We theoretically model the results based on the magnetic noise induced by the radicals and estimate the number of H_2_O_2_ molecules detected by the quantum sensor. Combining the peroxidase-mimic activities of the oxygenated NDs with its intrinsic quantum-sensing capability, we demonstrate that 10 nm fluorescent NDs can potentially be used as self-reporting H_2_O_2_ sensors with molecular-level sensitivity and nanoscale spatial resolution. These sensors will allow more precise detection of the H_2_O_2_ distribution in cells, which could contribute to earlier diagnosis of H_2_O_2_-related diseases as well as a better understanding of the role of H_2_O_2_ in stem cell biology, the immune response, cancer, and aging.

## Results and Discussion

### Characterization of NDs

**Figure 1:**
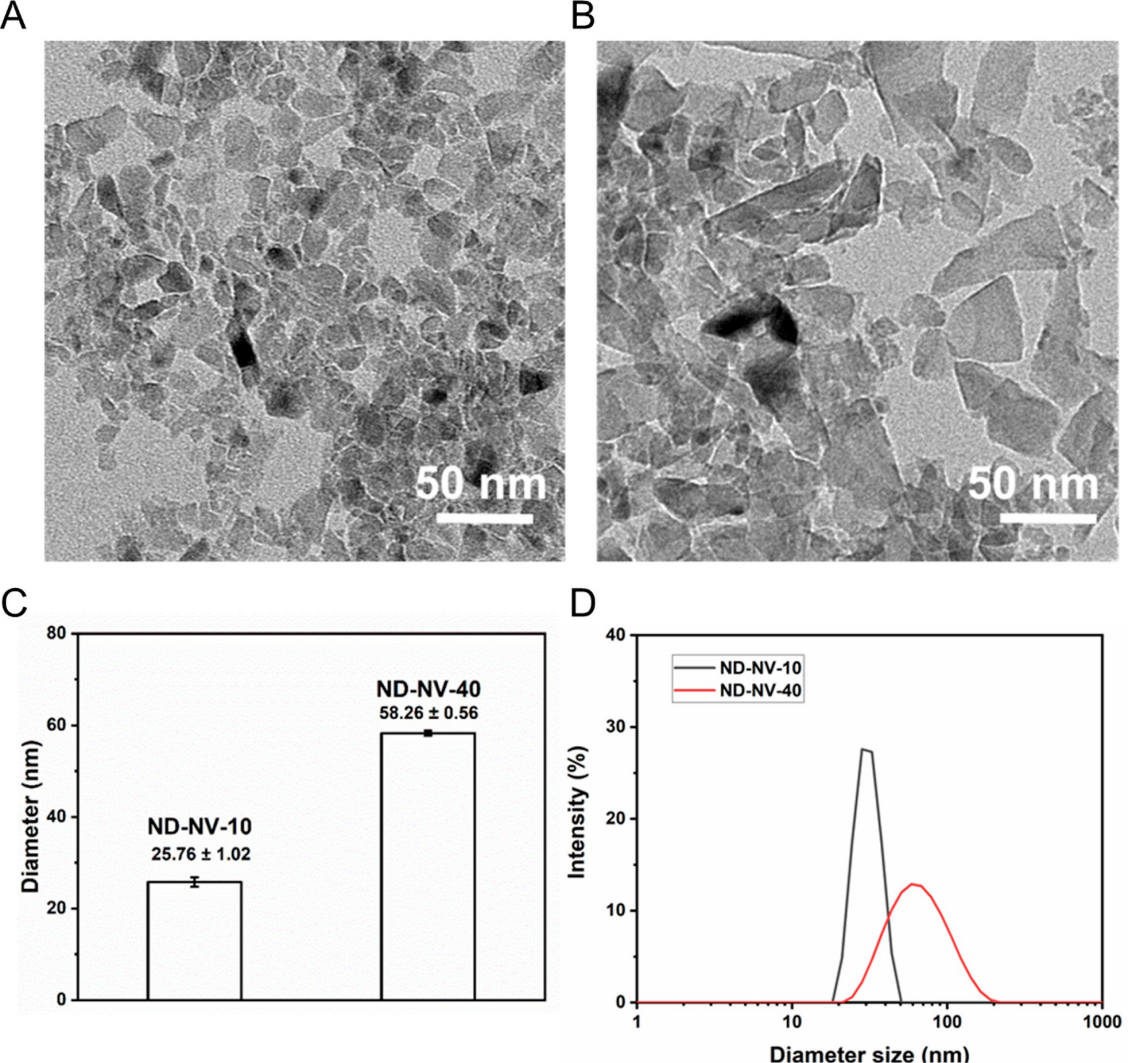
(A) TEM images of ND-NV-10 (scale bar = 50 nm); (B) TEM images of ND-NV-40 (scale bar = 50 nm); (C) hydrodynamic diameter of ND-NV-10 and ND-NV-40 measured by DLS, data presented as mean ± standard deviation, *n* = 3; and (D) hydrodynamic diameter distribution of ND-NV-10 and ND-NV-40 measured by DLS.

The ND samples, ND-NV-10 and ND-NV-40, used in this work, were purchased from Adamas Nanotechnologies, NC, USA. According to the manufacturer, they were produced by irradiating high-pressure high-temperature microdiamonds with 2–3 MeV electrons, annealing and milling the obtained microdiamonds, subsequently doing oxidative treatment in a mixture of nitric acid and sulfuric acid to obtain the oxygen-terminated surface, and separating the different size NDs by centrifugation.^34,35^ ND-NV-10 and ND-NV-40 were characterized using transmission electron microscopy (TEM) and dynamic light scattering (DLS) to analyze their shape, distribution, and morphology. As shown in Figure 1A,B, TEM images revealed that both ND-NV-10 and ND-NV-40 had an irregular, sharp, and inhomogeneous size distribution. The sizes of ND-NV-10 were in general much smaller than those of ND-NV-40. The histogram analysis of the TEM images of ND-NV-10 and ND-NV-40 revealed nanoparticle diameters of about 8.35 ± 4.24 and 27.87 ± 15.23 nm, respectively (Figure S1). The DLS results showed that the average hydrodynamic diameters of ND-NV-10 and ND-NV-40 in solution were 26 ± 1 and 58.3 ± 0.6 nm, respectively (Figure 1C,D). The measured hydrodynamic diameters agree with the TEM results, considering that the increase is due to the solvent shell. Both NDs showed a monomodal size distribution (Figure 1D), with the polydispersity index (PDI) of 0.255 for ND-NV-10 and 0.192 for ND-NV-40, respectively.

### Peroxidase-Mimicking Activity of ND-NV-10

**Figure 2:**
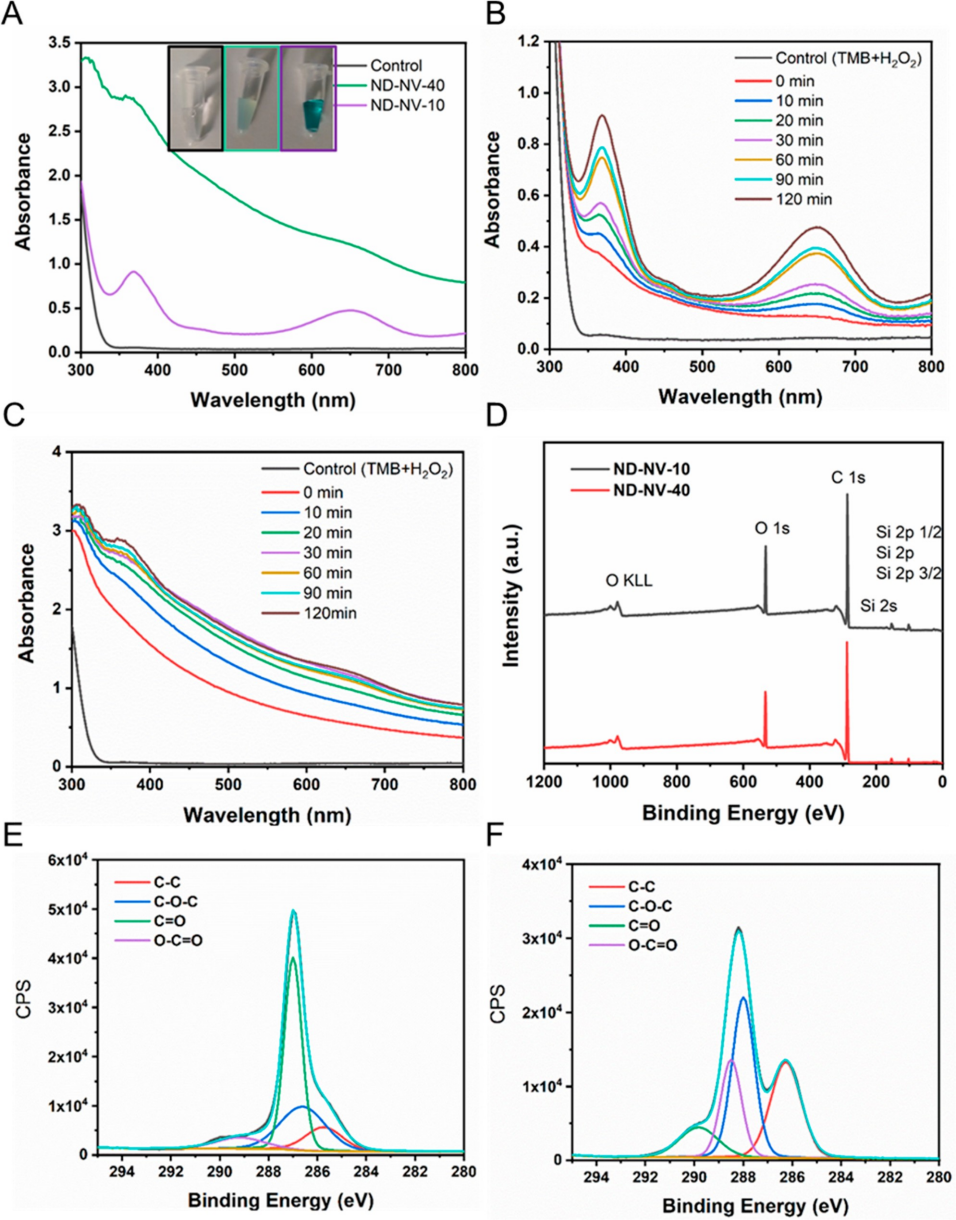
(A) Absorbance spectra of TMB in different reaction systems after 120 min; dark line: TMB + H_2_O_2_, green line: TMB + H_2_O_2_ + ND-NV-40, purple line: TMB + H_2_O_2_ + ND-NV-10. Inset: photos of H_2_O_2_ catalyzed by NDs in the presence of TMB, from left to right: TMB + H_2_O_2_, TMB + H_2_O_2_ + ND-NV-40, and TMB + H_2_O_2_ + ND-NV-10; (B) time-dependent absorbance spectra of TMB in the reaction system of TMB + H_2_O_2_ + ND-NV-10; (C) time-dependent absorbance spectra of TMB in the reaction system of TMB + H_2_O_2_ + ND-NV-40; (D) XPS spectra of ND-NV-10 and ND-NV-40; (E) C 1s core-level XPS spectra of ND-NV-10 (aqua lines) and corresponding fit (black lines); (F) C 1s core-level XPS spectra of ND-NV-40 (aqua lines) and corresponding fit (black lines).

To confirm the peroxidase-mimicking activity of ND-NV-10 and ND-NV-40, we used TMB, the most commonly used substrate for probing peroxidase acitivity.^36^ Generally, peroxidases promote the generation of hydroxyl radicals (HO^•^), which oxidize TMB to produce its diimide form that is blue. By measuring the absorbance spectra using a UV–vis spectrometer, we monitored the catalytic activity of the NDs. As shown in Figure 2A, compared to the control solution (TMB + H_2_O_2_), both samples with dispersed NDs (ND-NV-10 and ND-NV-40) showed a distinct blue color. The presence of the blue color directly indicated the catalytic activity of the NDs. Interestingly, the solution of ND-NV-10 displayed a much deeper blue coloration than ND-NV-40 of the same particle concentration, indicating a higher catalytic activity of the smaller NDs. The kinetic of the catalytic activity was studied by recording the absorbance peak at 652 nm as a function of the reaction time. As shown in Figure 2B, for ND-NV-10, we observed a distinct absorbance peak at 652 nm within 10 min of reaction time. Furthermore, the absorbance revealed a linear dependence up to a reaction time of 120 min(Figure S2). In contrast, the absorbance peak of ND-NV-40 (Figure 2C) was only observed after a reaction time of 120 min. These results further proved the higher catalytic activity of the smaller ND-NV-10 nanoparticles. Due to the production process of NDs (ball-milling of larger micronized diamond and centrifugation), ND-NV-40 also contains small-sized nanoparticles that might affect the catalytic activity. Therefore, small NDs were removed from ND-NV-40 by 5 times’ centrifugation at 12,000 rpm, as shown in the TEM image (Figure S3A). The hydrodynamic diameter increased from 58.3 ± 0.6 to 101.2 ± 0.3 nm due to the removal of small NDs (Figure S3B); the PDI of ND-NV-40 before and after 5 times’ centrifugation was 0.192 and 0.203, respectively, which shows no significant narrowing. Very weak catalytic activity was still observed (Figure S3C). In order to showcase the relevance of our results for cellular studies, we have assessed the catalytic activity of ND-NV-10 in biological buffers, Dulbecco’s phosphate-buffered saline (DPBS, pH = 7) and DPBS with 10% fetal bovine serum (FBS) that include proteins, electrolytes, lipids, carbohydrates, hormones, enzymes, and other undefined constituents to assess the influence of the more complex biological environment on the catalytic activity of ND-NV-10. The catalytic activity of ND-NV-10 (Figure S4) has still been retained under these conditions, which supports their potential future usage for in-cell sensing.

The marked difference in the catalytic activity of ND-NV-10 and ND-NV-40 could be attributed to the ND surface groups. Recent reports suggest that the catalytic activities of NDs are due to the carbonyl and/or carboxyl groups at the ND surface. X-ray photoelectron spectroscopy (XPS) was applied to quantify the ND surface groups and the corresponding XPS spectra are shown in Figure 2D. In Figure 2E,F, we show the high-resolution C 1s core-level XPS spectra of ND-NV-10 and ND-NV-40, respectively. The spectra were fitted with four Gaussian–Lorentzian curves with peaks centered at around 285.75, 286.60, 287.00, and 289.18 eV, assigned to the C–C bond,^37,38^ C–O–C bond,^38,39^ C=O bond,^40^ and O–C=O bond.^38,39^ The corresponding ratios of peak areas in ND-NV-10 were 13.10% for C–C groups, 29.23% for C–O–C groups, 49.67% for C=O groups, and 8.00% for O–C=O groups. In ND-NV-40, the corresponding ratios of peak areas were 30.13% for C–C groups, 37.03% for C–O–C groups, 20.88% for C=O groups, and 11.96% for O–C=O groups (Table S1). The overall percentage of C=O groups and O–C=O groups in ND-NV-10 was notably higher than in ND-NV-40, which might explain the higher catalytic activity of the smaller NDs. Moreover, the percentage of the O–C=O groups in ND-NV-40 was higher than that in ND-NV-10, indicating that ND-NV-40 may have a more negative zeta potential, which was in accordance with the measured zeta potential values of −25.9 ± 0.2 mV for ND-NV-10 and −31.0 ± 1.6 mV for ND-NV-40 (Figure S5).

DFT Calculations for the Understanding of the Catalytic Activity

**Figure 3:**
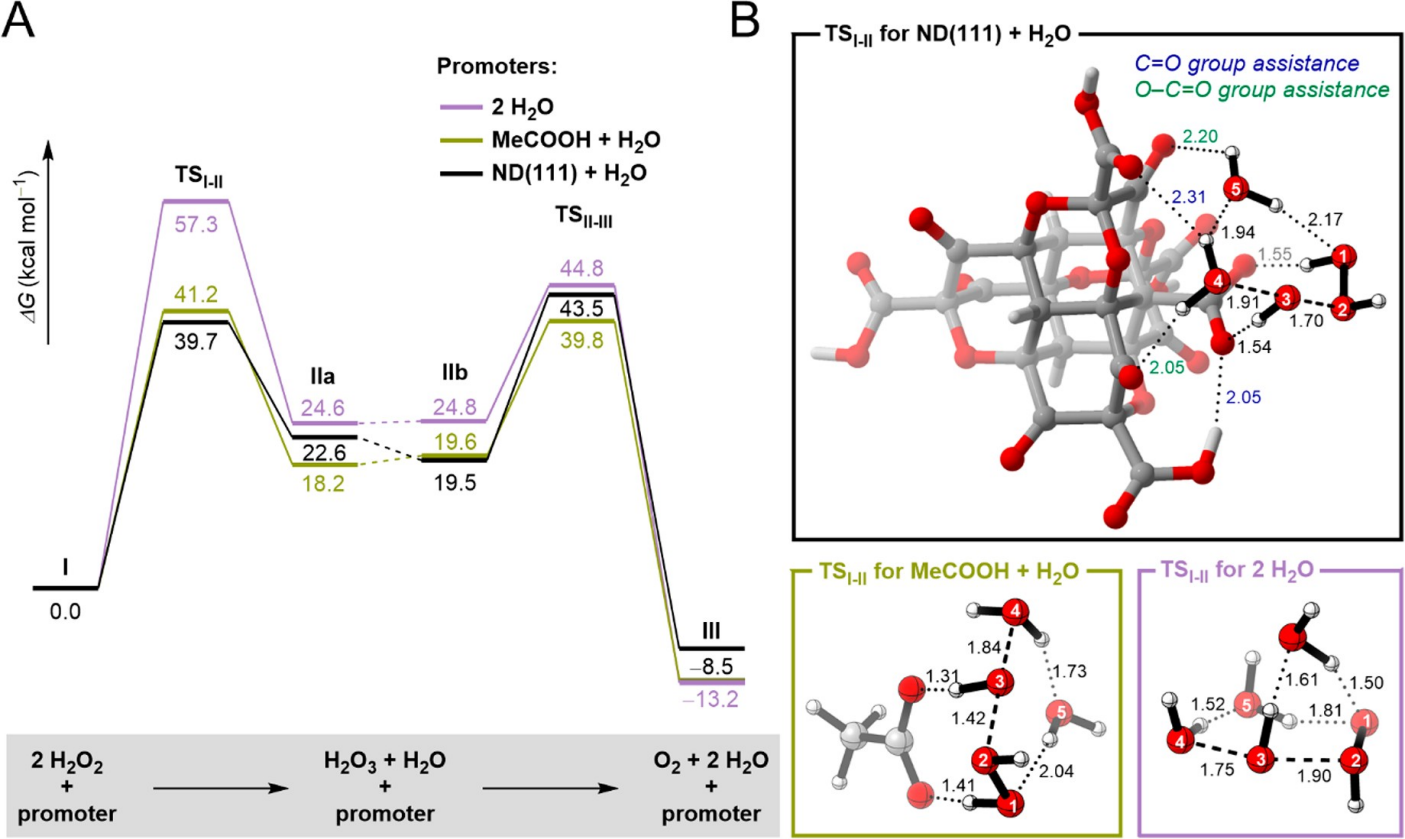
(A) Gibbs free energy profile for the decomposition reaction of H_2_O_2_ hydrogen peroxide promoted by different species. DFT calculations were performed at the M06-2X/6-31G(d) level of theory (energy values in kcal mol^–1^). (B) Transition-state geometries for the formation of the H_2_O_3_ radical for each promoter (selected distances in Å).

To further understand the role of NDs in the decomposition of H_2_O_2_, we performed DFT calculations at the M06-2X/6-31G(d) level of theory. The mechanism of the decomposition was assumed to occur in two steps via the reaction of two molecules of H_2_O_2_ to form H_2_O_3_ (^•^OH + ^•^O_2_H) radicals and H_2_O followed by the formation of O_2_ and H_2_O (Figure 3). We determined the reaction profile for three different promoters: (i) two molecules of water, (ii) one molecule of acetic acid and one molecule of water, and (iii) one molecule of ND(111) and one molecule of water. First, the calculated Gibbs free energies of activation for the two steps using two explicit water molecules were 57.3 and 44.8 kcal mol^–1^, respectively, which were in accordance with those reported by Tsuneda and Taketsugu.^41^ Next, to evaluate the efficacy of the O–C=O groups as promoters, we calculated the reaction profile after replacing one molecule of water by one molecule of acetic acid. Remarkably, the activation barriers decreased to 41.2 and 39.8 kcal mol^–1^, respectively, suggesting that carboxylic acid groups facilitate the decomposition of H_2_O_2_. Finally, we performed the calculations using model ND(111), which was designed based on the functional groups detected by XPS. The calculated energies’ activation barriers for the decomposition of H_2_O_2_ were similar to that of acetic acid. Furthermore, the analysis of the transition-state geometries for the first step (TS_I–II_, formation of the H_2_O_3_ radical) suggested that not only O–C=O groups but also C=O groups contribute to the hydrogen bonding network around the H_2_O_2_ molecules, stabilizing the transition-state structure and supporting the hypothesis that these groups control the catalytic efficiency of NDs.

### Investigation of the Molecular Scale Peroxidase Activity at the Single ND Level

**Figure 4:**
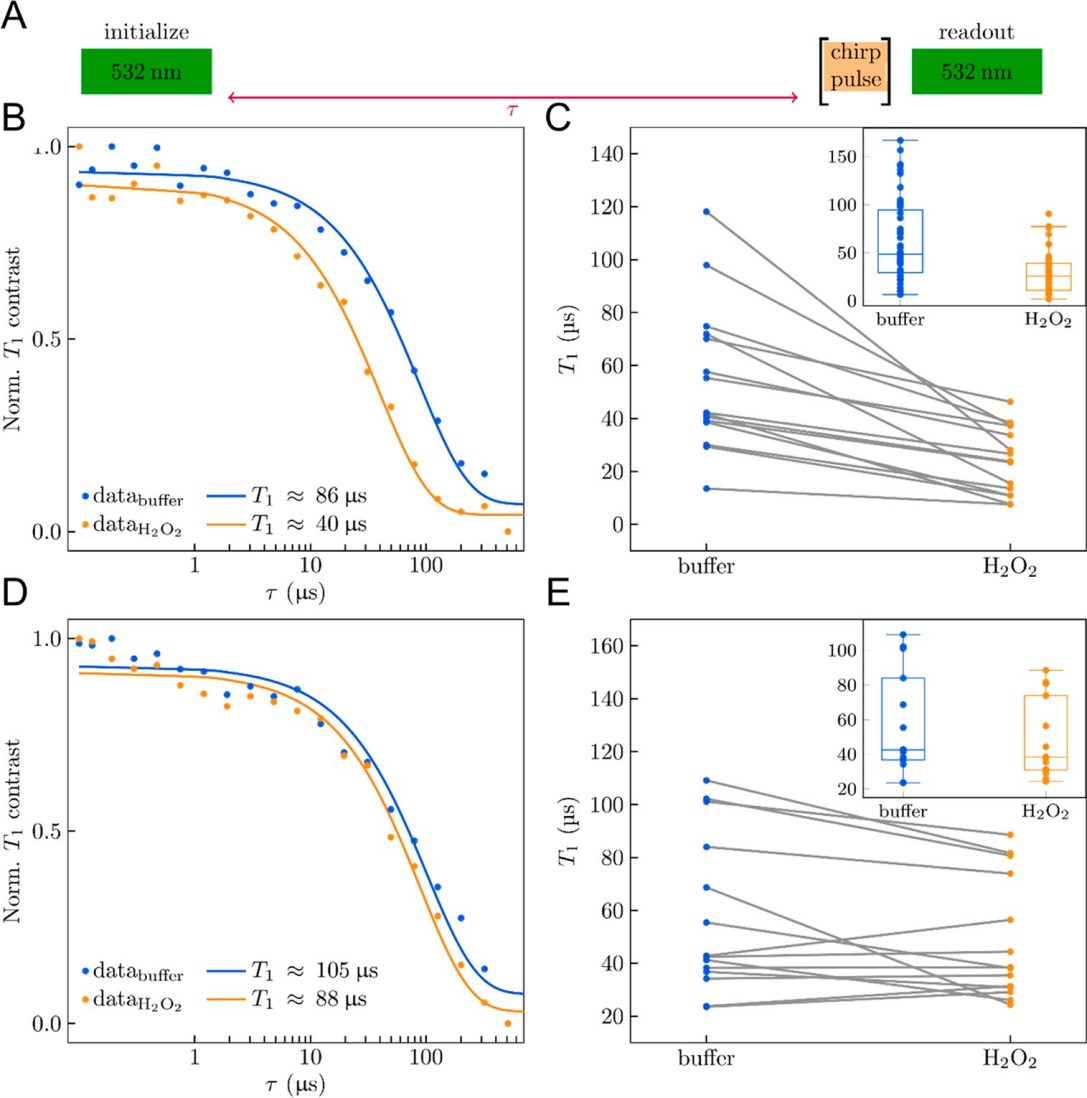
(A) Schematic presentation of pulse sequence for measuring the *T*_1_ time of the NV center. (B) Typical *T*_1_ relaxation curve of NV in ND-NV-10 in pH 4 acetate buffer (blue, dots) and with the addition of H_2_O_2_ (orange dots) solution. The solid lines are the single exponential fit to the measured data. (C) Comparison of the *T*_1_ relaxation time of 15 ND-NV-10 nanoparticles. The gray lines connect the individual ND measurements. Inset: box-and-whisker plot showing the distribution of *T*_1_ time (*N* = 44). (D) Typical *T*_1_ relaxation curve of NV in ND-NV-40 in pH 4 acetate buffer (blue, dots) and with the addition of H_2_O_2_ (orange dots) solution. The solid lines are the single exponential fit to the measured data. (E) Comparison of the *T*_1_ relaxation time of 14 ND-NV-40 nanoparticles. The gray lines connect the individual ND measurements. Inset: box-and-whisker plot showing the distribution of *T*_1_ time.

To investigate the molecular scale catalytic activity of individual NDs, we performed *T*_1_ relaxometry measurements on the NV quantum sensors. The radicals produced from H_2_O_2_ by the peroxidase activity of the NDs causes a fluctuating magnetic field noise in the vicinity of the NDs. This magnetic field noise can be measured by the NV center inside the NDs, which serves as a nanoscale signal transducer that converts the magnetic field fluctuations into a measurable optical signal.^42^ To measure the peroxidase activity of the NDs by quantum sensing, we first immobilized the NDs on a cleaned glass slide with a lithographically patterned microwave antenna. We placed a silicone gasket (cell well volume ∼30 μL) on top of the glass slide to confine the analyte in the subsequent measurements. As the NDs showed a high catalytic activity at pH = 4, we applied ∼5 μL of the acetate buffer solution (pH = 4), and the silicone well was covered with a glass slide to avoid evaporation. The *T*_1_ time was then measured on single isolated NDs. To study the peroxidase activity of the NDs, we applied ∼5 μL of 100 mM H_2_O_2_ solution and measured the *T*_1_ time on the same NDs as before (Figure S6). The pulse scheme for measuring the *T*_1_ time of the NVs is shown in Figure 4A. The *T*_1_ time was determined by first initializing the NV in the *m*_s_ = 0 state by using a green laser pulse. Following a variable waiting time τ, the NV spin state was read out using a subsequent laser pulse. The *T*_1_ time measured using this all-optical relaxometry technique is prone to optical anomalies such as charge-state switching of the NVs. Hence, to measure the *T*_1_ time due to magnetic noise, we applied an additional linear chirp pulse to invert the population from *m*_s_ = 0 to *m*_s_ = ±1 sublevels before readout. We then subtracted the data set to remove the common mode noise (see the Supporting Information). In Figure 4B, we have shown a typical *T*_1_ measurement on the ND-NV-10 sample, without (blue) and with (orange) the addition of H_2_O_2_ solution. The measurement was repeated on different ND-NV-10 nanoparticles (Figure 4C). Here, the *T*_1_ times measured in acetate buffer (blue) are compared to the nanoparticles after the addition of H_2_O_2_ solution (orange) measured on 15 individual NDs (only 15 of the 44 data points are shown here for clarity; others are included in Figure S4). The inset of Figure 4C depicts a box-and-whisker plot of the *T*_1_ distribution (*N* = 44). We observed that the mean *T*_1_ time decreased from ∼63 to ∼30 μs in the presence of H_2_O_2_. From the *T*_1_ distribution (the corresponding *T*_1_ values are given in Supporting Information Table S3), it was evident that in the presence of H_2_O_2_, ND-NV-10 promoted the decomposition of H_2_O_2_ molecules, generating radicals, which led to the shortening of the NVs *T*_1_ time. Similar experiments were performed with 14 ND-NV-40 nanoparticles under the same conditions (Figures 4D,E). We observed only a small change in the *T*_1_ time with the addition of the H_2_O_2_ solution. As discussed earlier, the small responsivity of ND-NV-40 to H_2_O_2_ molecules could be attributed to both the size of the NDs (relatively bigger than ND-NV-10; therefore, the NVs are less sensitive to the surface noise) and the presence of fewer surface groups producing the radicals.

In order to validate the potential application of the ND-based H_2_O_2_ sensors for biological samples, we performed similar experiments at pH = 7 using DPBS buffer (Figures S7–S9). First, we plotted a typical *T*_1_ measurement on the ND-NV-10 sample without (blue) and with (orange) addition of H_2_O_2_ solution at pH 7 and the comparison of the corresponding *T*_1_ time of 15 different NDs (only 15 of the 45 data points are shown for clarity; see Figure S8). The inset of Figure S8B shows the box-and-whisker plot of the *T*_1_ distribution at pH 7 from 45 individual measurements (*N* = 45). Although the mean *T*_1_ time at pH 7 is considerably shorter than at pH 4 due to electric field fluctuations caused by ion exchange at the surface,^19^ we observed a clear decrease in the *T*_1_ time upon addition of H_2_O_2_. The mean *T*_1_ time decreased from ∼27 to ∼12 μs in the presence of H_2_O_2_, proving the catalytic activity of the ND-NV-10 sample at pH = 7, thus ascertaining the usefulness of the sensor for biological applications. Furthermore, we also explored the catalytic activity of the ND-NV-10 sample in simulated body fluid (SBF) to mimic the relevant biological environment (Figure S10). Also here, the *T*_1_ time decreased with the addition of the H_2_O_2_ solution (*T*_1,SBF_ ∼ 31 and *T*_1,H2O2_ ∼ 17 μs).

### Theoretical Simulation of Spin Relaxation Times

We can infer the concentration of H_2_O_2_ molecules from the reduction of the *T*_1_ time of the NV center due to the presence of H_2_O_2_. To estimate the number of H_2_O_2_ molecules detected by an NV center, we used a theoretical model to simulate the *T*_1_ spin relaxation time of the NV *m*_s_ = 0 electron spin state. The ^•^OH or ^•^O_2_H radicals in the vicinity of the NV center produce a fluctuating magnetic noise at the position of the NV center that shortens the spin relaxation time, from *T*_1_^buffer^ (the spin relaxation time without the ^•^OH or ^•^O_2_H radicals) to *T*_1_. Their relation is given by
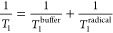


**Figure 5:**
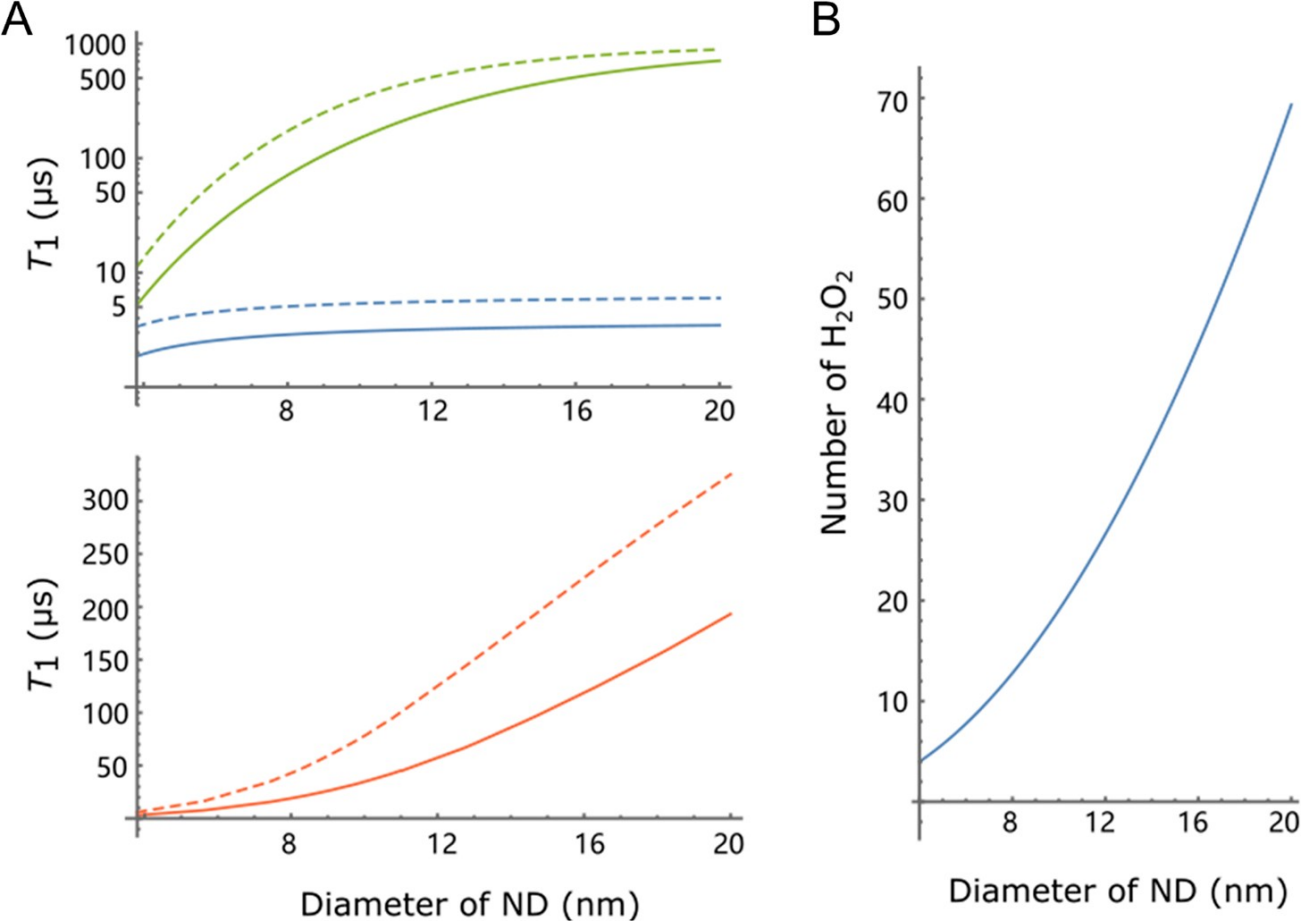
(A) Simulated spin relaxation times of an NV center for different diameters of NDs, before (dashed lines) or after (solid lines) the addition of H_2_O_2_ solution. The green lines correspond to the case where the NV center is located in the center of the ND and has the longest relaxation times. The blue lines represent the NV center that is close to the diamond surface with the shortest relaxation times. The red lines are the mean values for the randomly chosen position and orientations of the NV centers. The density (0.05/nm^3^) of OH radicals was chosen such that it reduces the spin relaxation times by ∼ 56% for a diamond diameter close to the average raw size of ND-NV-10. (B) Estimated number of H_2_O_2_ molecules within a distance of 1 nm to the diamond surface by using the density of OH radicals used in (A).

To calculate 1/*T*_1_^radical^, we modeled the ^•^OH or ^•^O_2_H radicals as an ensemble of randomly fluctuating spins with a volume density ρ, and we assumed that each ND has a spherical shape, in which an NV center is randomly located in the ND. Considering that the random locations of the NV could be very close to or far from the diamond surface, the assumption of a spherical shape provides a good approximation for the simulation and is also in accordance with the previous works.^29,43^ Because the NV center is not stable when it is very close to the surface, we introduced a constraint in the model that the NV center should be at least a 1 nm distance below the diamond surface. We considered that there were surface electrons at the ND surface, which made *T*_1_^buffer^ smaller for smaller NDs. The amplitude variance of the magnetic noise produced by the ^•^OH or ^•^O_2_H radicals, *B*_⊥_^2^ = Σ_*j*_*B*_⊥,*j*_^2^, is a sum of the terms due to each radical electron spin^29,43^
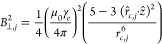
where μ_0_ is the vacuum permeability, γ_*e*_ is the electron gyromagnetic ratio, *ẑ* is the unit axis along the NV symmetry axis, and *r*_*c,j*_*r̂*_*c,j*_(with |*r̂*_^*c*,*j*_| = 1) is the position of the *j*-th ^•^OH or ^•^O_2_H radical relative to the NV center. The summation in *B*_⊥_^2^becomes an integral when we assume a volume density ρ for the radical electrons. Using a time correlation *B*_⊥_^2^*e*_*c*_^–|τ/τ|^ (*B*_⊥_^2^ being the amplitude variance and τ_*c*_ being the correlation time) for the fluctuating magnetic noise produced by the ^•^OH or ^•^O_2_H radicals, the increased decay rate due to the magnetic noise of the OH or O_2_H radicals is given by^43^
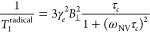
where ω_NV_ ≈ 2π × 2.87 GHz is the NV electron spin resonance frequency. We show the simulated spin relaxation times of *T*_1_^buffer^ (dashed lines) and *T*_1_ (solid lines) for the NV centers as a function of the ND sizes in Figure 5A, where the red lines were obtained by averaging the spin relaxation times over all possible positions and orientations of the NV center in the ND, while the green (blue) lines represent the results for the longest (shortest) spin relaxation times for a particular diamond size in the simulation. In performing the averaging, we randomly chose the position and orientation of each NV so that the NV is located within the allowed diamond sphere before the corresponding relaxation time is calculated. Because the effect of NV–NV coupling on the NV spin relaxation is similar to the effect of surface electron spin noise and the NVs have a low density, we ignored possible NV–NV coupling in the simulation when there are multiple NVs in a single ND. The average plot shown in Figure 5A was obtained by 10^5^ random NV configurations for a convergent Monte Carlo simulation. We have tuned the densities of the ^•^OH or ^•^O_2_H radicals and the surface electron spins at the diamond surface so that the ratio of the relaxation time *T*_1_/*T*_1_^buffer^ is approximately 44% as observed in the experiments (see the Supporting Information for more details). From the relaxation times, we could estimate the amount of H_2_O_2_ molecules detected by the NV center in the NDs (Figure 5B). For the ND-NV-10 nanoparticles, the change in the *T*_1_ relaxation time corresponds to a detection of about 20 H_2_O_2_ molecules. Note that this number corresponds to the highest number of H_2_O_2_ molecules that can be detected by the nanoparticles. In our experiment, we could detect ∼3 radicals within 10 s of integration time (see the Supporting Information for more details). In contrast to the traditional H_2_O_2_ detection, where a calibration curve needs to be measured first in most of the cases,^44,45^ our method is calibration-free. In addition, most work on the detection of H_2_O_2_ focuses on the detection limit of the concentration but ignores the required absolute number of H_2_O_2_ molecules and the volume of H_2_O_2_. In most of the cases, H_2_O_2_ solutions in the microliter range are used to achieve a nanomolar or even picomolar concentration detection limit. However, the absolute number of H_2_O_2_ required is still more than 10^5^.^46,47^

## Conclusions

In this study, we have shown that sub-10 nm oxygenated fluorescent NDs provide a high catalytic activity for the decomposition of H_2_O_2_ molecules. Due to the intrinsic quantum-sensing features of NV centers, these NDs could serve as self-reporters of locally produced radicals from H_2_O_2_ molecules. In addition, we have demonstrated the catalytic activity and the sensing ability of ND-NV-10 in complex environments mimicking biological media, such as DPBS (pH = 7), DPBS with 10% FBS including proteins, electrolytes, lipids, carbohydrates, hormones, enzymes, and other undefined constituents, and SBF, which supports their potential future usage for in-cell sensing. Moreover, until now, it has not been possible to distinguish H_2_O_2_ and other radicals present in cells. However, due to the difference of catalytic activity between ND-NV-40 and ND-NV-10, our method could potentially serve as a tool to differentiate H_2_O_2_ from other radicals. Combining the measured *T*_1_ reduction with theoretical simulation, we estimate that the nanoparticles decompose about 20 H_2_O_2_ molecules. To the best of our knowledge, this is the first demonstration of NDs as self-reporting sensors for any chemical species. Furthermore, this work establishes the local production and quantitative detection of H_2_O_2_ with molecular-level sensitivity (∼3 radicals) and nanoscale spatial resolution (∼500 nm^3^ or ∼500 × 10^–18^ μL). In contrast, the most sensitive methods reported so far can detect more than 10^5^ H_2_O_2_ molecules at a concentration of 1 pM and a volume of 1 μL.^46^ In addition, we have also demonstrated the molecular-level sensitivity of the ND sensor that could detect very low H_2_O_2_ concentrations (100 pM) with nanoscale spatial resolution (∼500 nm^3^ or ∼500 × 10^–18^ μL). Given the diverse functionalizability of the NDs, the sensor offers the potential to quantify intracellular and extracellular H_2_O_2_ produced by living cells. We expect to unravel the role of H_2_O_2_ in the process of DNA methylation as a possible application. By combining the simplicity and the specificity of the catalytic activity of the NDs, the sensor could be employed to detect H_2_O_2_ molecules in a range of complex and contaminant-prone samples such as whole blood, the food industry, environmental analysis, and fuel cells.

## Supporting Information Available

Details of materials, instruments, and experimental procedures; DFT calculations; NV center spin relaxation time measurement; simulation of spin relaxation times; and sensitivity estimation (PDF)
