# Supplementary material for: Detection of Few Hydrogen Peroxide Molecules Using Self-Reporting Fluorescent Nanodiamond Quantum Sensors
Source: J Am Chem Soc. 2022 Jun 23;144(28):12642–51. doi: 10.1021/jacs.2c01065 (PMC9305977; doi:10.1021/jacs.2c01065)
Supplement: Supplementary file 1 — ja2c01065_si_001.pdf [file ja2c01065_si_001.pdf]

Supporting information

Detection of Few Hydrogen Peroxide Molecules Using Self-Reporting Fluorescent Nanodiamond Quantum Sensors

Yingke Wu†, Priyadharshini Balasubramanian†, Zhenyu Wang, Jaime A. S. Coelho, Mateja Prslja, Reiner Siebert, Martin B. Plenio, Fedor Jelezko, and Tanja Weil

† Y. W and P. B contributed equally

CONTENTS:

1. Experimental section

    1.1 Materials
    1.2 Methods
    1.3 Density functional theory (DFT) calculations
    1.4 NV center spin relaxation time measurement
    1.5 Simulation of spin relaxation times
    1.6 Sensitivity estimation

2. Tables and Figures

3. References

1. Experimental section

1.1 Materials

Nanodiamonds, ND-NV-10 and ND-NV-40 were purchased from Adámas Nanotechnologies; 3,3′,5,5′-tetramethylbenzidine solution (TMB, Liquid Substrate System for ELISA), hydrogen peroxide (H_2O_2) solution, sodium acetate and acetic acid were purchase from Sigma-Aldrich and were used without further purification, 3,3′,5,5′-tetramethylbenzidine powder was purchased from Thermofisher Scientific, H_2O was obtained from the Millipore purification system. Simulated Body Fluid was purchase from Biochemazone.

1.2 Experiments

Transmission Electron Microscopy (TEM)

One drop of a 0.1 mg/mL solution of ND-NV-10 or ND-NV-40 in MilliQ was placed onto an oxygen treated copper grid and dried at room temperature. A Jeol 1400 transmissions electron microscope was used to obtain bright field images

Dynamic Light Scattering (DLS)

500 µL of 0.1 mg/mL solutions of ND-NV-10 or ND-NV-40 in MilliQ were transferred into a borosilicate glass cuvette and measured at 25 °C with a 90° angle using a particle sizer (Malvern Zetasizer Nano-S90 (Nano series)). The hydrodynamic diameter distribution was presented as intensity. Zeta potential was measured at 25 °C with a Malvern Zetasizer Nano-S90 (Nano series).

UV-Vis Absorbance for catalytic activity analysis

50 µL of a 100mM H_2O_2 solution and 50 µL TMB solution were added in one of the wells of a 96-well microplate, and either100 µL H_2O, 100 µL 1 mg/mL ND-NV-10 or 100 µL 1mg/mL ND-NV-40 were added in. Absorbance scans from 300 to 800 nm were measured at the time point of 0 min, 10 min, 20 min, 30 min, 60 min, 90 min, and 120 min using a Tecan Spark 20M microplate reader.

X-Ray Photoelectron Spectroscopy

10 μL of a 1 mg/mL ND-NV-10 or ND-NV-40 aqueous solutions were deposited on a silicon substrate and dried at room temperature. XPS was conducted using a Kratos Axis UltraDLD spectrometer 3 (Kratos, Manchester, England) using an Al Kα excitation source with a photon energy of 1487 eV. The data was acquired in the hybrid mode using a 0° take-off angle, defined as the angle between the surface normal and the axis of the analyzer lens. Detailed region XP spectra were collected with setting analyzer pass energy at 80 eV, and a linear background was subtracted for all peak quantifications. The peak areas were normalized by the manufacturer supplied sensitivity factors and surface concentrations were calculated using CasaXPS software. C 1s high-resolution spectra were collected with analyzer pass energy of 20 eV. Neutralizer was always used during spectra collection.

1.3 Density functional theory (DFT) calculations

Density functional theory (DFT) calculations were performed using the Gaussian 09 software package[1] and structural representations were generated with CYLview20.[2] All the geometry optimizations were carried out using the Minnesota hybrid meta-GGA functional M06-2X and valence double-zeta 6-31G(d) basis set. All of the optimized geometries were verified by frequency computations as minima (zero imaginary frequencies) or transition states (a single imaginary frequency corresponding to the desired reaction coordinate). The free energy values presented in Figure 3 in main text and Table S2 were derived from the electronic energy corrected by using the thermal and entropic corrections based on structural and vibration frequency data.

1.4 NV center spin relaxation time measurement

The spin relaxation time (T_1) of NV centers in nanodiamonds are performed in a customized confocal microscope. We use a 532nm laser to excite the NV center. The laser power (measured before the objective) is fixed at 50 μW for all the experiments. The laser is focussed onto the sample using an oil-immersion objective (Olympus UPlanSApo 60x oil, N.A. = 1.35). The fluorescence from the NV center is collected through the same objective and detected using an avalanche photodiode (APD). We use a 650nm longpass filter in front of the APD to eliminate the excitation laser and minimize the detection of NV^0.

The pulse sequence for the T_1 measurement is shown in the main text (Fig 4). It consists of a laser pulse (10μs long) to polarize the NVs in the $m_s = 0$ state. After a variable waiting time τ, the subsequent laser pulse is used to read the spin state of the NV center. We use fluorescence photons detected in the first 500 ns of the laser pulse to constitute the signal. This all-optical T_1 measurement is prone to charge state fluctuation of the NV, which would manifest as a decrease in NV fluorescence as a function of time. To measure the actual T_1 time of the NV we use an additional control sequence with a linear chirp microwave pulse to invert the population from the $m_s = 0$ to $m_s = \pm1$ state. The frequency range for the linear chirp microwave pulse is from 2850 MHz to 2950 MHz and is generated using an arbitrary waveform generator. The duration of the chirp and the Rabi frequency is adjusted to achieve the adiabatic passage condition. The T_1 measurements without inversion pulse is subtracted from the T_1 measurements with inversion pulse to obtain the spin relaxation time due to magnetic noise. The experiment is repeated several times with a total acquisition time of 10 minutes. The resulting T_1 data is fitted with a monoexponential decay function.

To measure the T_1 time statistics of the nanodiamonds, we used cleaned glass slide with a lithographically patterned MW structure. We placed a silicone gasket (cell well volume ~30μL) on top of the glass slide to confine the nanodiamonds and the analyte solution in the subsequent measurements. Next, we added ~10 μL of 10 μg/mL sample into the cell well and the sample was dried for several hours under ambient conditions. During the T_1 measurement, we added ~5 μL of buffer (acetate buffer for pH 4 and DPBS for pH 7) and covered the silicone well with a glass slide to avoid evaporation. The T_1 time is measured as described above on selected isolated fluorescence spots (with fluorescence counts between 400-600 kcts/s). Following the T_1 measurements in the buffer, we added ~5 μL of 100 mM H_2O_2 solution and repeated the T_1 measurement on the same fluorescent spots as before.

Furthermore, we also performed the similar relaxometry experiments with smaller H_2O_2 concentration and observe a change in T_1 time even for 100 pM concentration (Figure S11). In order to explore the catalytic activity of the ND-NV-10 sample in biological samples, we performed similar relaxometry experiments in simulated body fluid (SBF) to mimic the relevant biological environment (Figure S10). We observe a clear decrease in the T_1 time ($T_{1,\,\mathrm{SBF}}$ ~ 31 μs and $T_{1,\,\mathrm{H_2O_2}}$ ~ 17 μs) with the addition of H_2O_2 solution. Although the change in T_1 time is slightly

smaller than in the controlled environment (T1 change with SBF ~ 45% compared to ~56% or higher with acetate or DPBS buffer), we see a clear decreasing trend of T_1 time with H_2O_2 solution.

Similar experiments were performed with 14 ND-NV-40 nanoparticles under the same conditions (Figure S8 A and D). We observed only a small change in T_1 time with the addition of the H_2O_2 solution. As discussed earlier, the small responsivity of ND-NV-40 to H_2O_2 molecules could be attributed to both the size of the NDs (relatively bigger than ND-NV-10, therefore the NVs are less sensitive to the surface noise) and the presence of fewer surface groups producing the radicals. Some of the nanodiamonds in both samples NV-NV-10 and NV-NV-40 showed an increase in T_1 time with the addition of H_2O_2. The behaviour was prevalent in nanodiamonds with short NV T_1 time (< 20 μs). Since the effects of optical artefacts are removed, we attribute the T_1 increase to the removal of certain surface groups due to the oxidative activity of the H_2O_2. All the confocal and quantum sensing experiments are performed with Qudi software.[3]

1.5 Simulation of spin relaxation times

Without the OH or O_2H radicals, the $m_s = 0$ spin relaxation is determined by the intrinsic NV relaxation and the electron spin noise at the surface of the nanodiamond, with the relaxation rate given by

$$\frac{1}{T_1^{\text{buffer}}} = \frac{1}{T_1^{\text{i}}} + \frac{1}{T_1^{\text{noise}}}.$$

Here we take the intrinsic NV relaxation time $T_1^{\text{i}} = 1$ ms, a value similar to the ones of NVs in bulk diamond. The relaxation rate $1/T_1^{\text{noise}}$ due to the noisy electrons at diamond surface is calculated in a way similar to that of the OH or O_2H radicals.

The correlation times τ_c for the noisy electrons at diamond surface and for the OH or O_2H radical spins are obtained using the results of Tetienne *et al.*[4]. We have

$$\tau_c = \left(\frac{\mu_0 \gamma_e^2}{16\pi} \frac{(8\pi\rho)^{1/2}}{r_{\text{min}}^{3/2}} + R_{\text{vib}}\right)^{-1},$$

where $R_{\mathrm{vib}} = 50$ GHz takes into account the intrinsic vibrational spin relaxation.[3] The parameter related to the minimum allowed distance among the electron spins is 0.15 nm for the noisy electrons at diamond surface and is 0.2 nm for the OH or O_2H radicals. We assume a small thickness (0.1 nm, which is much smaller than the diamond sizes) for the surface electrons layer on top of the diamond surface. To obtain ~56% reduction of NV spin relaxation times observed in the experiments, the noisy electrons at the diamond has a density of 0.18/nm^3 while the OH or O_2H radicals has a density of 0.05/nm^3 for the simulation.

1.6 Sensitivity estimation

The smallest number of radicals that can be detected is given as[4],

$$N_{min} = \frac{1}{P\sqrt{\Delta t}} d_0^4 \, f(\sigma)$$

Where $P = \frac{C}{2\pi}\sqrt{\frac{A\,\mathcal{R}\,T_{int}}{2\,e}}$ and $f(\sigma) = \frac{\sigma^{1/4}(\sigma+B)^{3/2}}{\sigma+3B}$. In our experiment, $C \sim 0.2$, $\mathcal{R} \sim 300\ kcts/s$, $T_{int} \sim 600\ ns$.

2. Tables and Figures

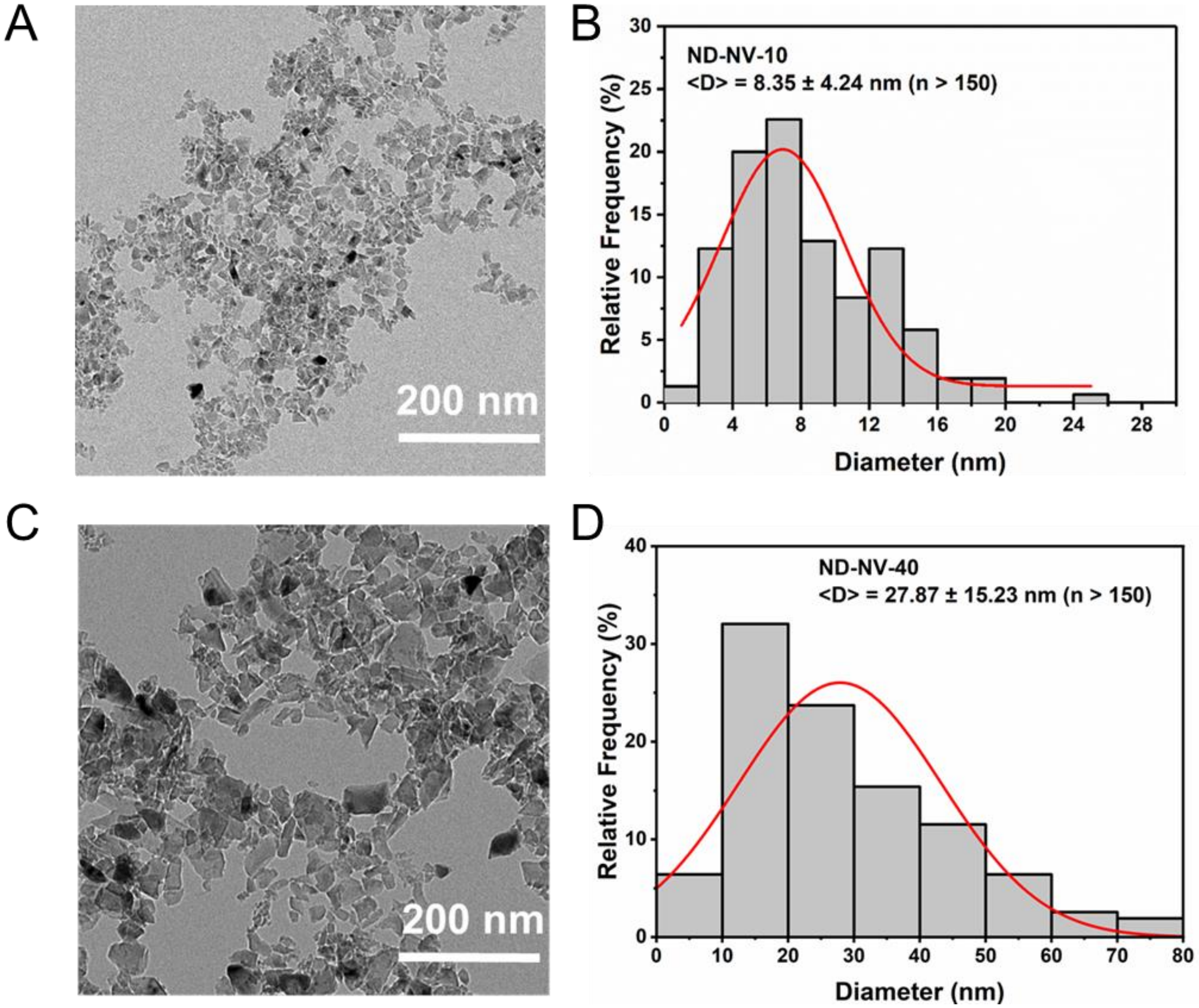

Figure S1. (A) TEM image ND-NV-10 (scale bar: 200 nm), (B) The size distribution histogram of ND-NV-10. (C) TEM image ND-NV-40 (scale bar: 200 nm), (D) The size distribution histogram of ND-NV-40.

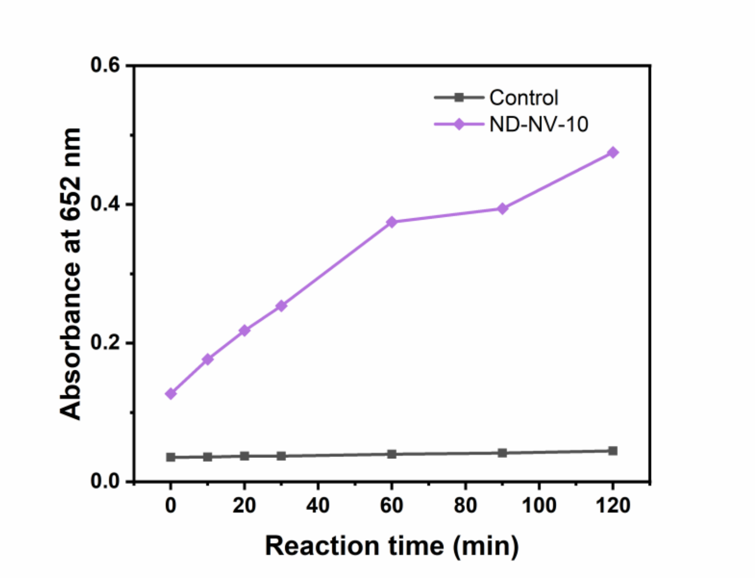

Figure S2. Time-dependent absorbance changes at 652 nm in the present of ND-NV-10.

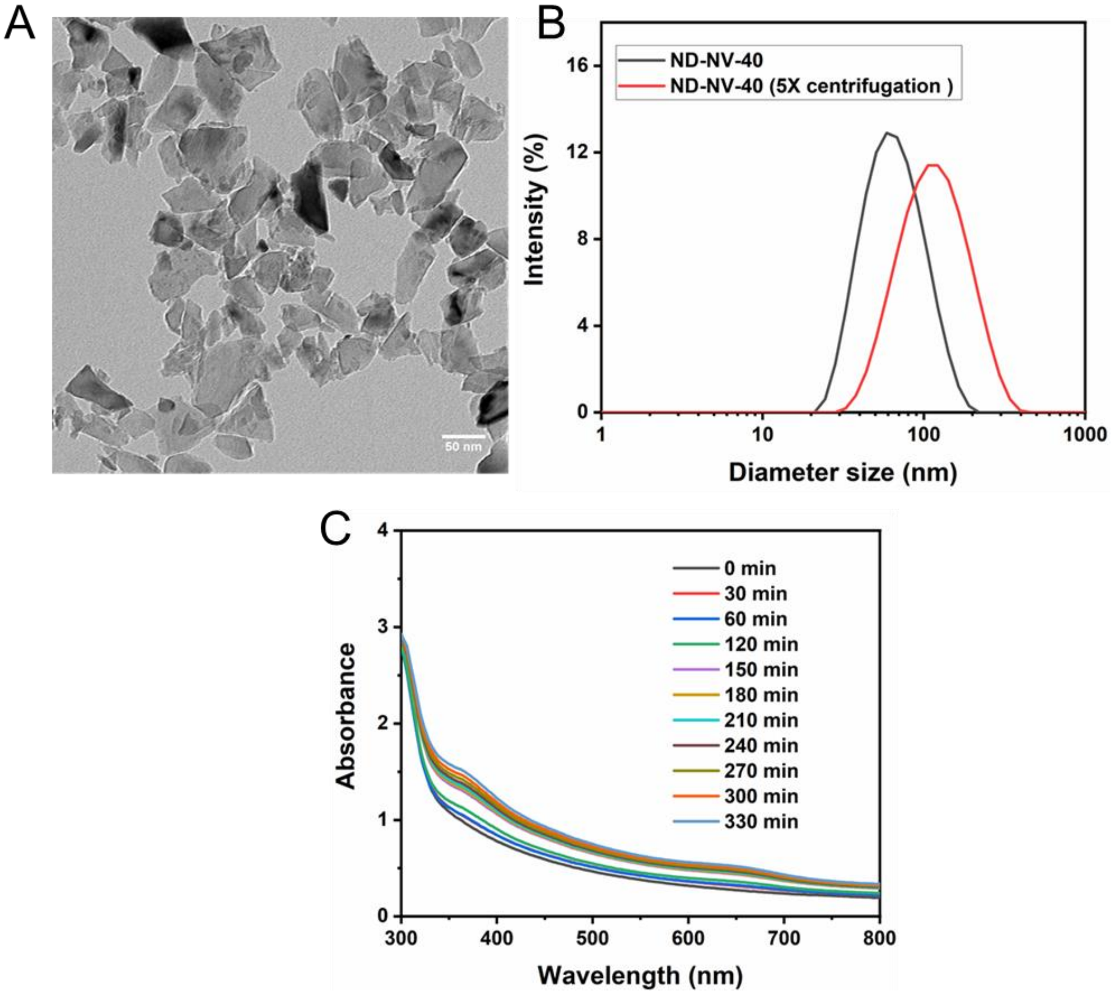

Figure S3. (A) TEM images of ND-NV-40 after 5 times fractionation by centrifugation at 12,000 rpm to remove the small sized NDs in the supernatant (scale bar = 50 nm); (B) Hydrodynamic diameter distribution of ND-NV-40 and ND-NV-40 after after 5 times fractionation by centrifugation at 12,000 rpm to remove the small sized NDs in the supernatant by DLS; (C) Time-dependent absorbance spectra of TMB in the reaction system of TMB + H_2O_2 + ND-NV-40 (after removing the small sized NDs).

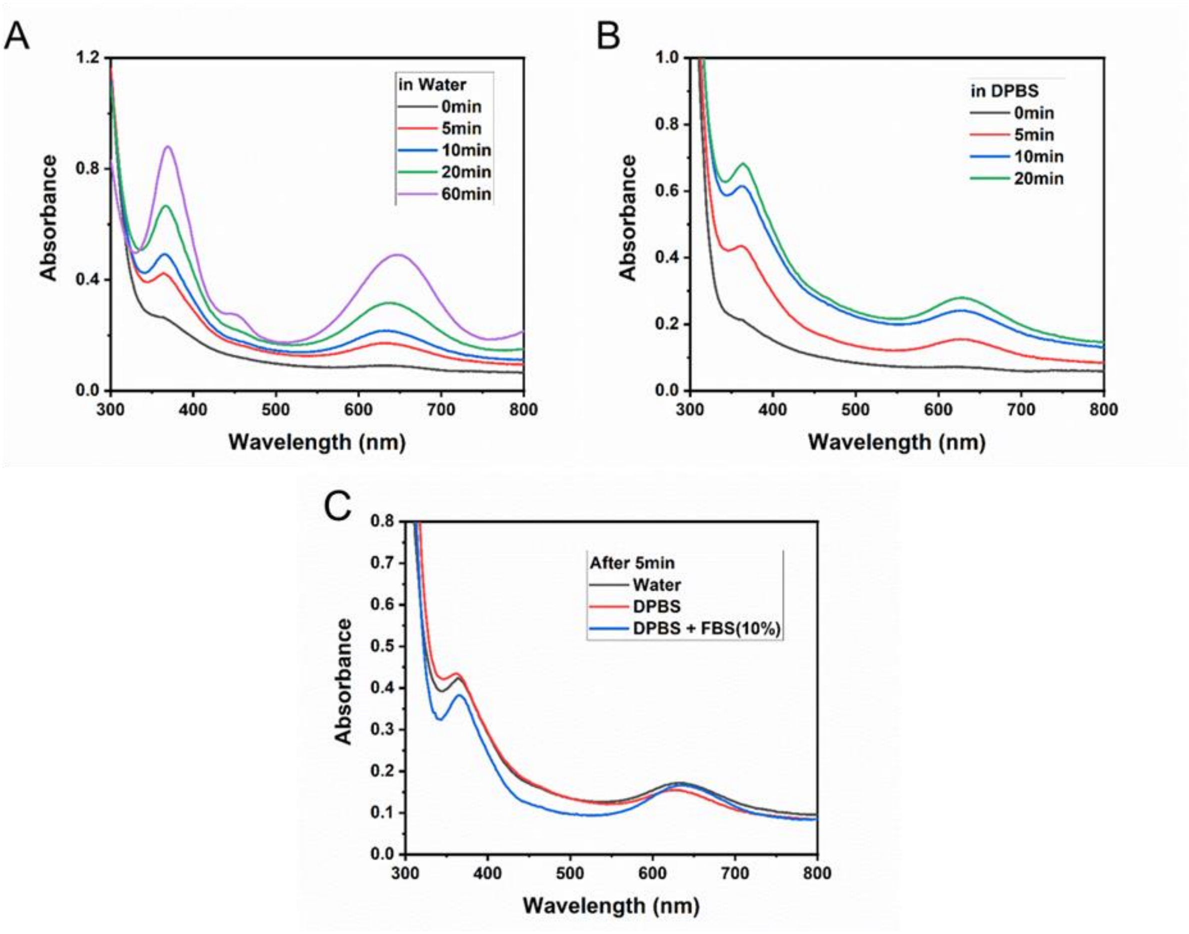

Figure S4. Time-dependent absorbance spectra of TMB in the reaction system of TMB + H_2O_2 + ND-NV-10 in water ((pH = 7.3) (A), and DPBS buffer ((pH = 7.05)(B), (C) Absorbance spectra of TMB in different reaction systems after 5 min. Dark line: TMB + H_2O_2 + ND-NV-10 in water, red line TMB + H_2O_2+ ND-NV-10 in DPBS, blue line: TMB + H_2O_2+ ND-NV-10 in DPBS containing 10% FBS.

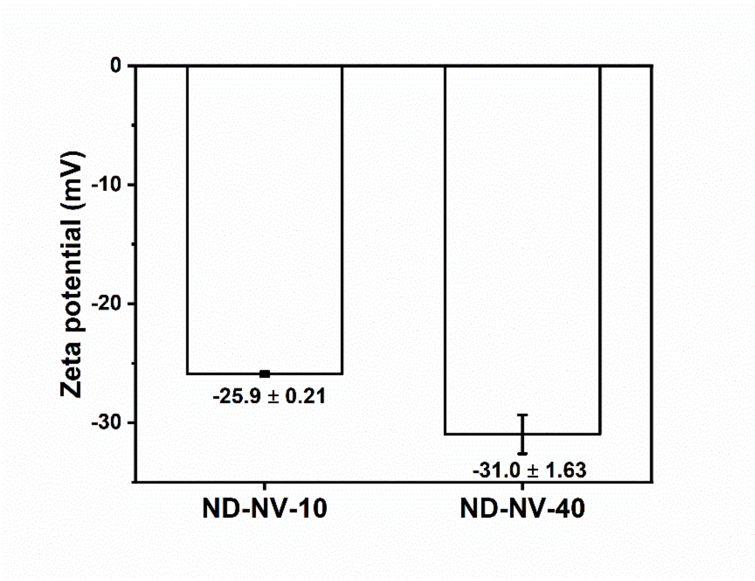

Figure S5. Zeta-potential of ND-NV-10 and ND-NV-40, data presented as mean ± SD, n = 3.

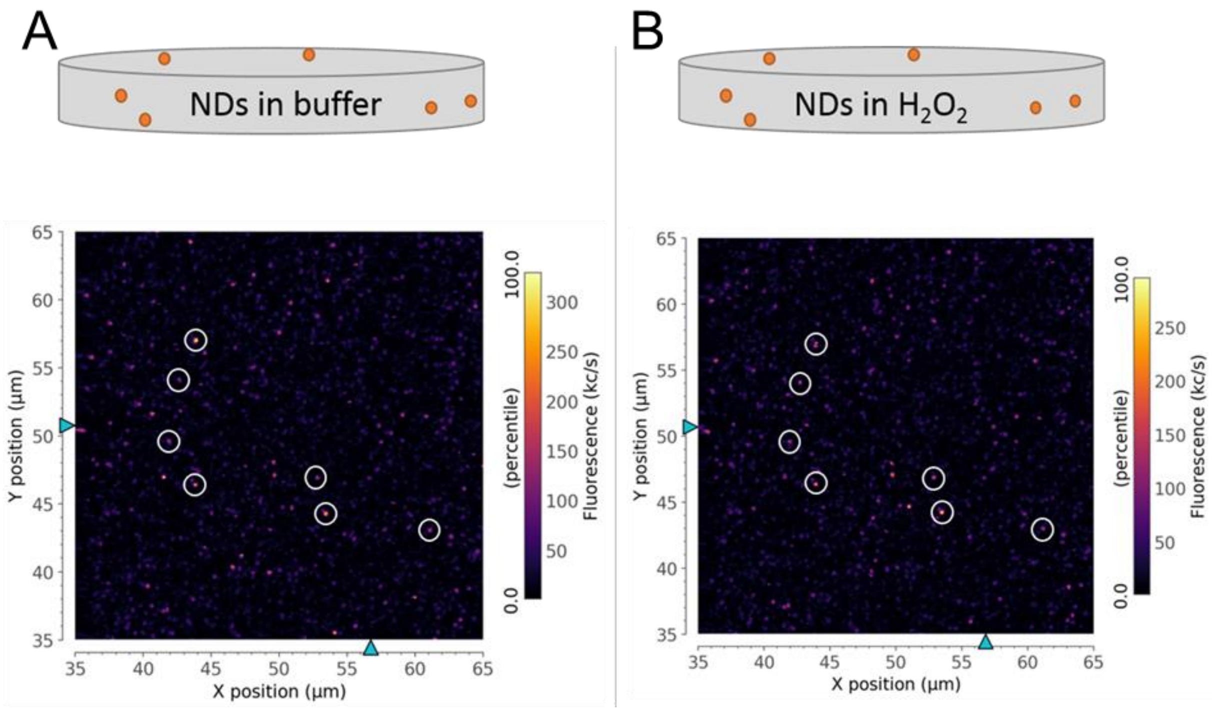

Figure S6. Confocal images of ND-NV-10 before (A) and after (B) adding H_2O_2.

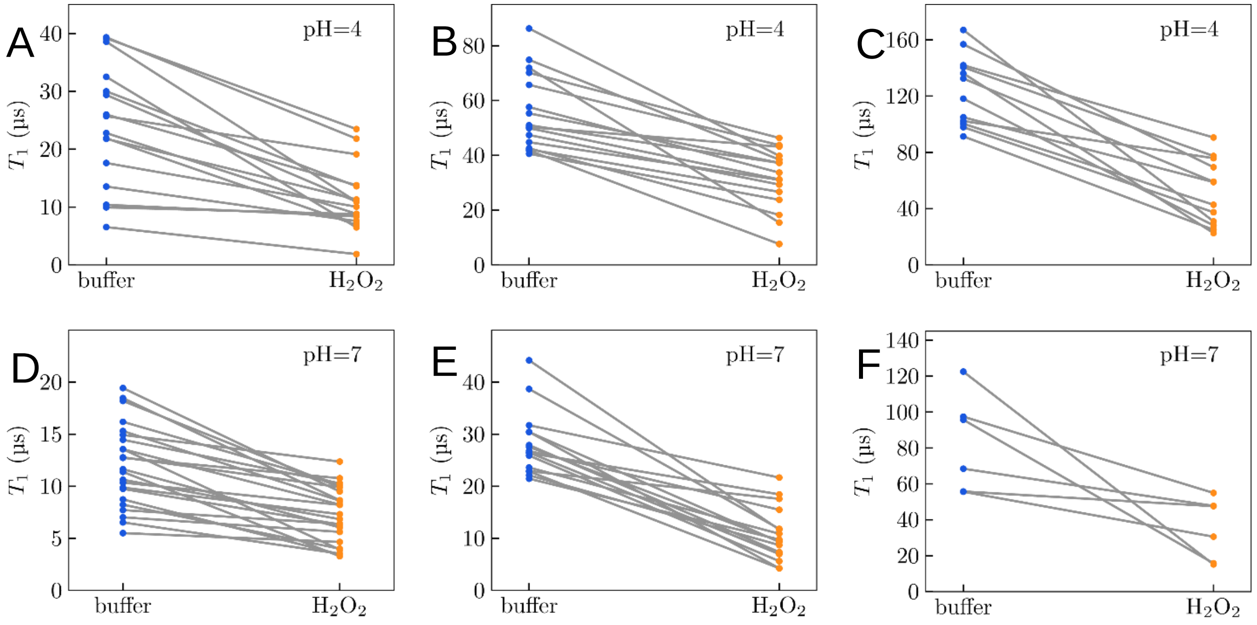

Figure S7. Comparison of the T_1 relaxation time of ND-NV-10 nanoparticles at pH 4 (A-C) and at pH 7 (D-F). Blue dots represent the measured T_1 time with the buffer (acetate buffer for pH 4 and DPBS for pH 7) and the orange dots represent the T_1 time with the addition of H_2O_2 solution The grey lines connect the individual ND measurements.

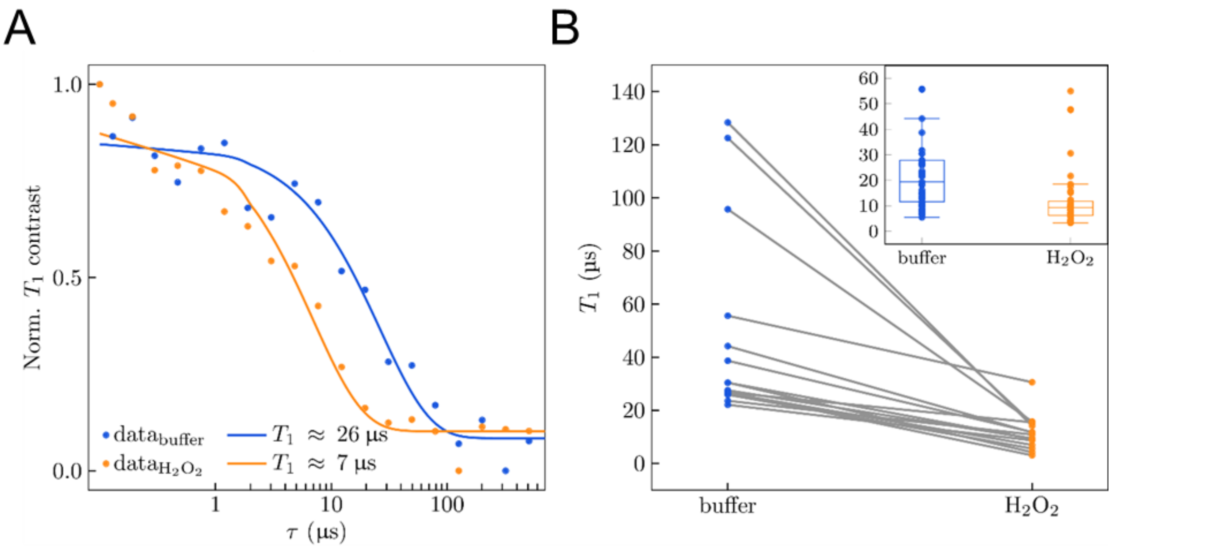

Figure S8. (A) Typical T_1 relaxation curve of NV in ND-NV-10 at pH 7 DPBS buffer (blue, dots) and with the addition of H_2O_2 (orange dots) solution. The solid lines are single exponential fit to

the measured data. (B) Comparison of the T_1 relaxation time of 15 ND-NV-10 nanoparticles. The grey lines connect the individual ND measurements. Inset: Box-and-whisker plot showing the distribution of T_1 time (N = 45).

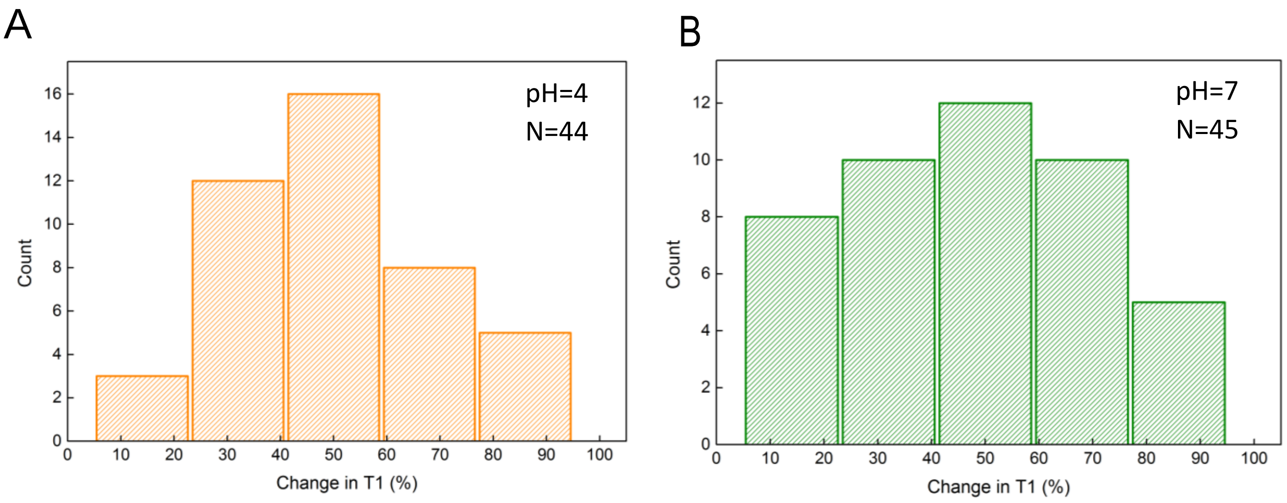

Figure S9. Histogram of change in T_1 time (($T_{1,\text{ buffer}}$ - $T_{1,\text{ H2O2}}$)/ $T_{1,\text{ buffer}}$) for pH 4 (A) and pH 7 (B).

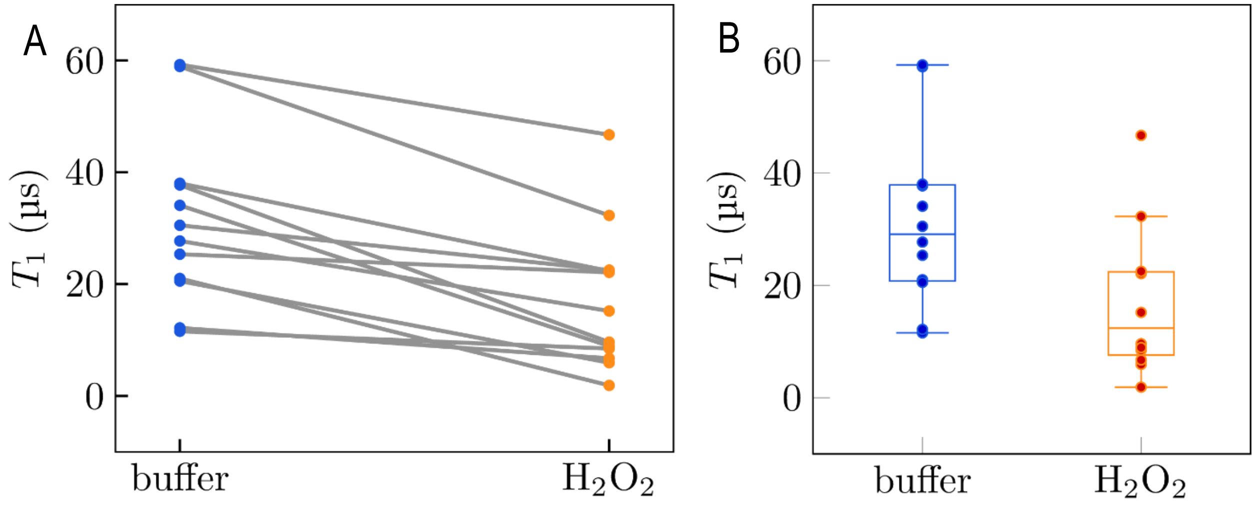

Figure S10. (A) Comparison of the T_1 relaxation times of ND-NV-10 nanoparticles in simulated body fluid (SBF) buffer. The grey lines connect the individual ND measurements. (B) Box-and-

whisker plot showing the distribution of T_1 time for 12 different nanodiamonds. Mean T_1 time is given in Table S3.

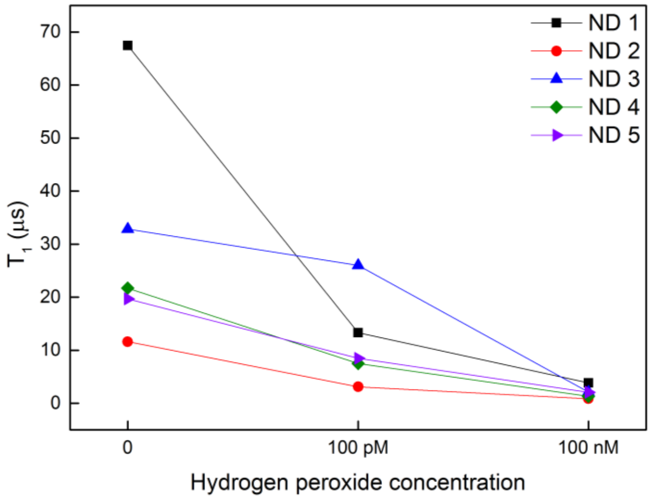

Figure S11. Measured T_1 time of 5 different nanodiamonds of sample ND-NV-10 after the addition of 100 pM and 100 nM H_2O_2 concentration. The measurements were performed at pH 7.

Table S1. XPS C1s core level fitting results of the presence of different functional groups on the surfaces.

		**C-C**	**C-O-C**	**C=O**	**O-C=O**
**ND-NV-10**	Binding Energy(eV)	285.75	286.60	287.00	289.18
	Area (%)	13.10	29.23	49.67	8.00
**ND-NV-40**	Binding Energy(eV)	286.26	288.00	288.50	289.83
	Area (%)	30.13	37.03	20.88	11.96

Table S2. Calculated energies and the geometry

Promoter		SCF energy (Hartree)	Free energy correction (Hartree)	Imaginary frequency (*i*cm^{-1})
**2 H_2O**	**I**	−455.748810681	0.074552	
	**TS_{I-ii}**	−455.658725336	0.075844	545.3
	**IIa**	−455.711574894	0.076579	
	**IIb**	−455.711569278	0.076838	
	**TS_{II-iiI}**	−455.673332106	0.070506	1444.0
	**III**	−455.765981539	0.070684	
**MeCOOH + H_2O**	**I**	−608.353936339	0.109608	
	**TS_{I-ii}**	−608.284035824	0.105363	509.9
	**IIa**	−608.322273983	0.106875	
	**IIb**	−608.320264792	0.107186	
	**TS_{II-iiI}**	−608.284008919	0.103070	1462.6
	**III**	−608.368293236	0.102891	
**ND(111) + H_2O**	**I**	−2990.81199942	0.358543	
	**TS_{I-ii}**	−2990.74911635	0.358869	444.2
	**IIa**	−2990.77505116	0.357670	
	**IIb**	−2990.78186028	0.359426	
	**TS_{II-iiI}**	−2990.73814749	0.353936	1543.6
	**III**	−2990.82052324	0.353504	

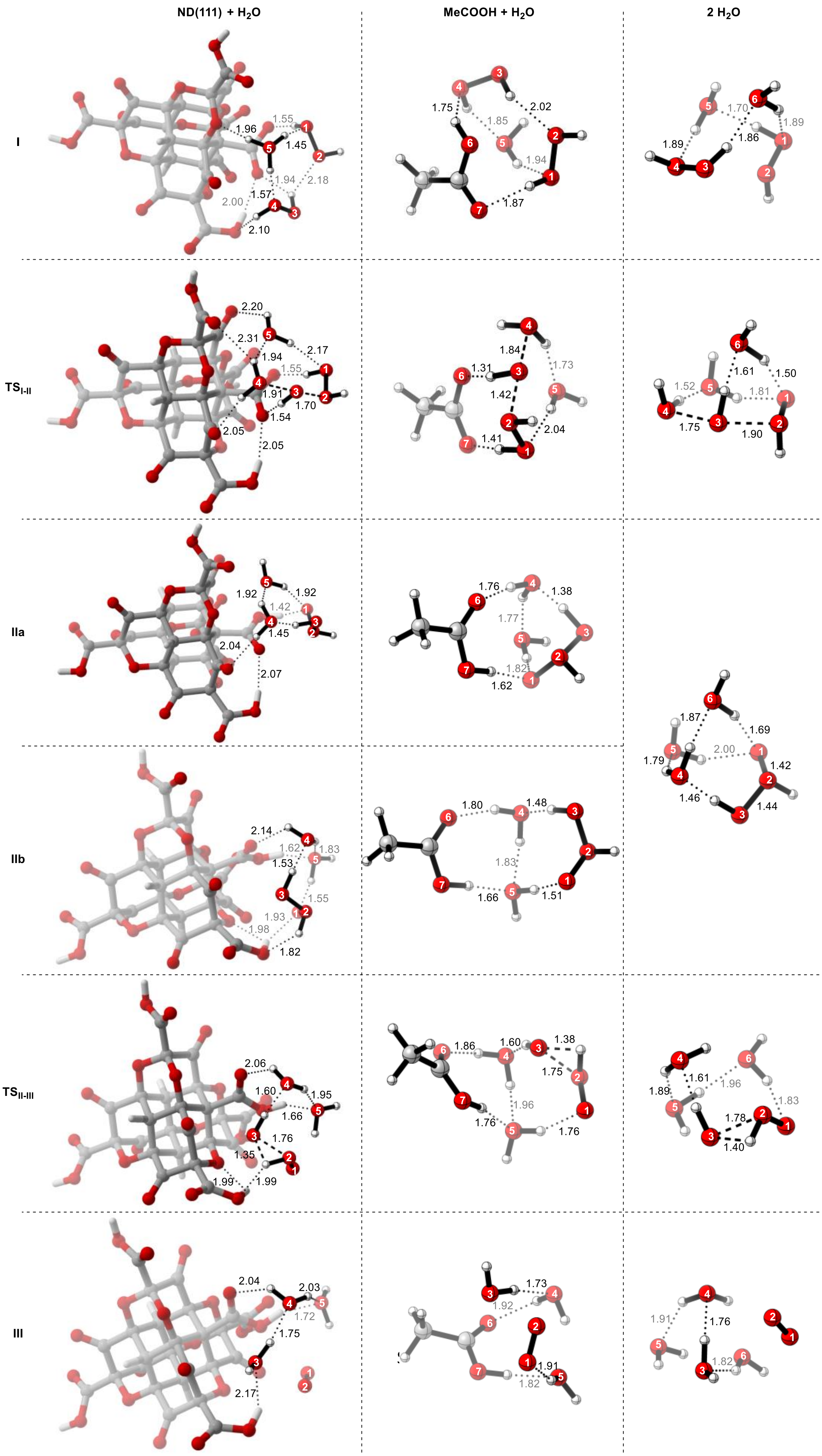
ND(111) + H_2O
MeCOOH + H_2O
2 H_2O
I
TS_{I-II}
IIa
IIb
TS_{II-III}
III

Figure S12. Optimized structures (M06-2X/6-31G(d)) for the reaction of H_2O_2 decomposition.

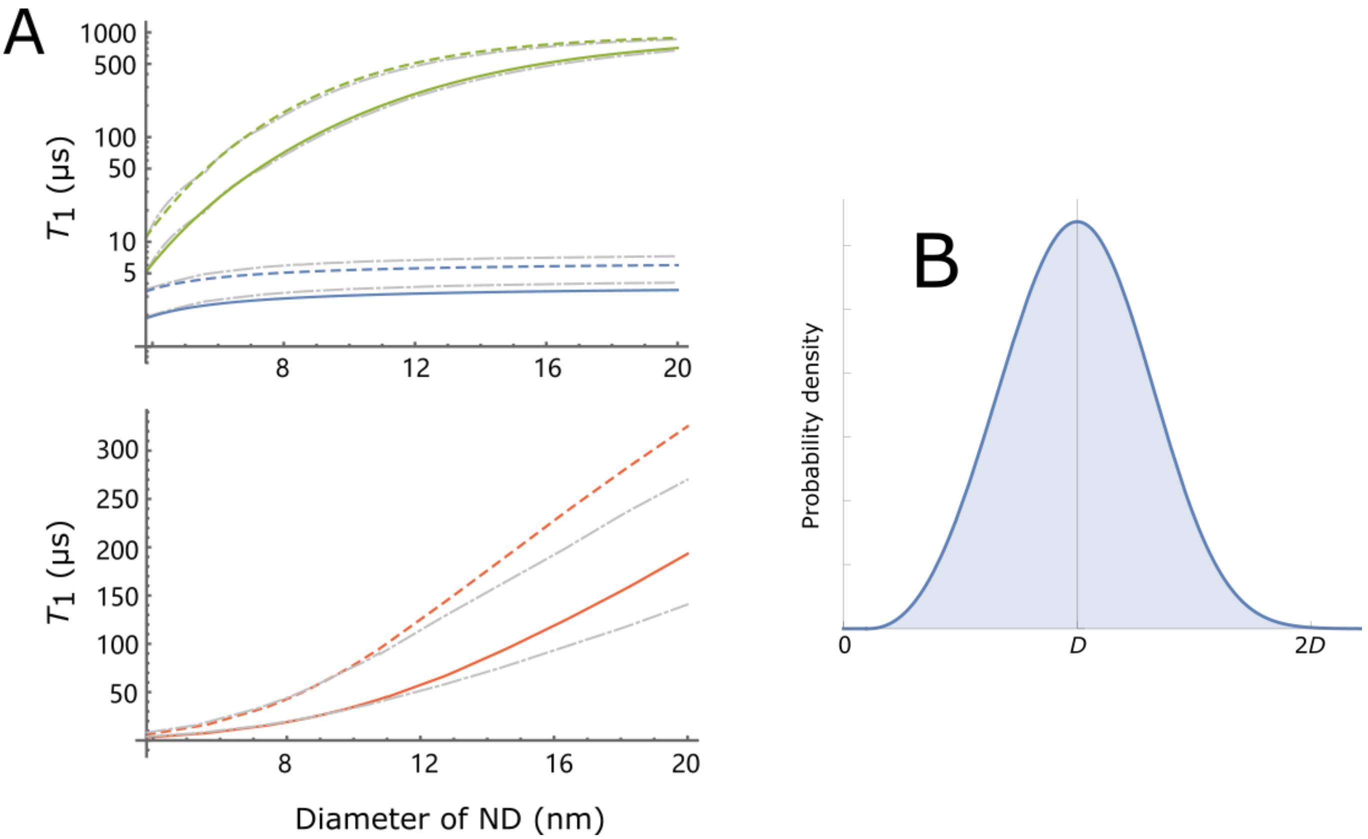

Figure S13. (A) The gray dot-dashed lines are the spin relaxation times of NV centers, averaging over different elliptical shapes of NDs. The results are similar to the ones of spherical NDs (green, blue, red lines that are also shown in Fig. 5 of the main manuscript). Here, the averaging is performed by randomly chosen the length *L* of a principal axis of the elliptical ND following a Weibull distribution, while the other two principal axes are fixed to the diameter *D* of the ND, i.e., the x-axis of the plots in (A). The orientations of the elliptical NDs are also random with respect to the NV symmetry axis. (B) The Weibull distribution used in (A) has a mean value *D* and has a standard deviation of 0.3*D*. This distribution has a probability density function (PDF) similar to a normal distribution but the PDF is zero when the length *L* is smaller than 0.

Table S3. Statistics of NV T_1 time.

Sample	Sample size (N)	Before H_2O_2 addition		After H_2O_2 addition	
		Mean (μs)	SD (μs)	Mean (μs)	SD (μs)
**ND-NV-10 (pH = 4)**	44	62.887	43.516	29.67	21.216
**ND-NV-10 (pH = 7)**	45	27.156	25.216	12.298	11.523
**ND-NV-10 (SBF)**	12	31.413	15.564	16.896	12.976
**ND-NV-40**		57.506	30.034	48.553	23.042

3. References

1. Frisch, M. J.; Trucks, G. W.; Schlegel, H. B.; Scuseria, G. E.; Robb, M. A.; Cheeseman, J. R.; Scalmani, G.; Barone, V.; Mennucci, B.; Petersson, G. A.; Nakatsuji, H.; Caricato, M.; Li, X.; Hratchian, H. P.; Izmaylov, A. F.; Bloino, J.; Zheng, G.; Sonnenberg, J. L.; Hada, M.; Ehara, M.; Toyota, K.; Fukuda, R.; Hasegawa, J.; Ishida, M.; Nakajima, T.; Honda, Y.; Kitao, O.; Nakai, H.; Vreven, T.; Jr., J. A. M.; Peralta, J. E.; Ogliaro, F.; Bearpark, M.; Heyd, J. J.; Brothers, E.; Kudin, K. N.; Staroverov, V. N.; Kobayashi, R.; Normand, J.; Raghavachari, K.; Rendell, A.; Burant, J. C.; Iyengar, S. S.; Tomasi, J.; Cossi, M.; Rega, N.; Millam, J. M.; Klene, M.; Knox, J. E.; Cross, J. B.; Bakken, V.; Adamo, C.; Jaramillo, J.; Gomperts, R.; tratmann, R. E.; Yazyev, O.; Austin, A. J.; Cammi, R.; Pomelli, C.; Ochterski, J. W.; Martin, R. L.; Morokuma, K.; Zakrzewski, V. G.; Voth, G. A.; Salvador, P.; Dannenberg, J. J.; Dapprich, S.; Daniels, A. D.; Ö. Farkas; Foresman, J. B.; Ortiz, J. V.; Cioslowski, J.;Fox, D. J. GAUSSIAN 09 (Revision D.01), Gaussian, Inc.: Wallingford CT, 2009.
2. CYLview20; Legault, C. Y., Université de Sherbrooke, 2020 (http://www.cylview.org).
3. Binder JM, Stark A, Tomek N, Scheuer J, Frank F, Jahnke KD, Müller C, Schmitt S, Metsch MH, Unden T, Gehring T. Qudi: A modular python suite for experiment control and data processing. *SoftwareX*. **6**, 85 (2017).
4. Tetienne, J-P., T. Hingant, L. Rondin, A. Cavaillès, L. Mayer, G. Dantelle, T. Gacoin, J. Wrachtrup, J-F. Roch, and V. Jacques. Spin relaxometry of single nitrogen-vacancy defects in diamond nanocrystals for magnetic noise sensing. *Phys. Rev. B* **87**, 235436 (2013).
